# Too much charity?

**DOI:** 10.1038/s44319-025-00542-1

**Published:** 2025-07-29

**Authors:** Frank Gannon

**Affiliations:** QIMR Berghofer, Brisbane, QLD Australia

**Keywords:** Cancer, Economics, Law & Politics, Genetics, Gene Therapy & Genetic Disease

## Abstract

Charities are a conduit to channel generosity into noble causes, often to meet an urgent health need. But they need to address duplication, waste and inefficiency to avoid cynicism among donors and dwindling funds.

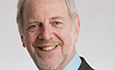

Recently, I was at a sport event with a friend where we could buy the electronic equivalent of a raffle ticket to support a charity dedicated to mental health. The prize was 50% of the evening’s donations. My friend was lucky and won a few thousand dollars, which he collected from a professional lottery company that presumably was paid by the charity to organize the event. There may have been other costs, such as advertising, personnel, preparation of annual accounts and perhaps even the right to be in the stadium. When all of these are added up, there may have been only a few hundred dollars left to support the mental-health cause of the raffle. It seemed like a lot of effort for a small return that will have only a minimal impact.

I could name five other local charities that focus on mental health, but I had not heard of the one at the sports event. It raises the question about why so many of these small and unknown charities exist. Primarily, they respond to two types of need. The first, in the general area of health, is to address some significant health problem. Many countries have large charities that provide targeted funding for research into and prevention of major diseases such as cardiovascular disease, mental health issues and various cancers. When added up, that might define about 20 health causes for charities to exist. There are 600,000 charities in Germany, 1.5 million in France, 1.8 million in the USA, 3 million in India and 185,000 in the UK. In a defined area such as cancer, there are 620 dedicated charities in the UK, hundreds in Australia and over a thousand major cancer charities worldwide that are part of the Union for International Cancer Charities. These divergent figures suggest that the definition of a charity can be rather loose to include anything from all not-for-profit entities in some countries to a smaller number of more tightly defined charities in others. As charity status comes with tax benefits, the rules related to that can give rise to these different numbers in different countries. Nonetheless, they all share the goal of having an impact on some aspect of society, and there are a lot of them.

There is also a dynamic in the number of charities as some fade away while new ones are being established. This leads to the second type of need that they address. News of a family tragedy or a local event helps to raise awareness about a certain disease or a lack of social support, and the community feels that they need to “do something”. However, some of these small charities eventually realize that the scale of support needed is orders of magnitude greater than what the local effort can deliver. Over time, fatigue from volunteers and donors sets in and the fundraising comes to a silent halt.

When I was involved in fundraising for the institute that I directed, I had a golden rule based on best practice for charities: when the cost of an event was more than one-third of the funds raised, it should be discontinued. We had signed up to an international group franchise that arranged a bike and a walk event to fund cancer research. There were fixed costs as part of the contract and the franchise did a great job with advertising and logistics which included having roads closed and policed and overnight camping and food. The participants contributed generously, but each year the income diminished as donor fatigue set in and the percentage that it cost to run the event grew uncomfortably. We eventually ended both events, to the surprise of regulars. But it was the right thing to do. Nonetheless, it was a great success while it lasted. The participants could see and relate to the researchers and trust the organization, and they acted as surrogate fundraisers among friends and colleagues. The collective effort of hundreds of donors yielded significant support for the institute. In a sense, we were providing them with a conduit for their generosity.

To achieve this success, at least for a couple of years, is more challenging for someone with a sick child who is trying to raise funds to support research on the disease that the child is suffering from. Even if the illness is common and other charities already fund research related to it, the personal drive to “do something” wins out and another small charity is created. Eventually, numerous small charities care for the same cause, and the chances of raising enough money to make an impact are slim despite their best efforts. In fact, inefficiency and duplication reduce the credibility of such small charities. While heart-tugging posters with children or women—men do not “sell” as well—who suffer the consequences of chemotherapy, are hard to ignore, it can become counterproductive when only a small percentage of a small amount raised is the outcome to make an impact on the disease. The suggested guide of a maximum of 30% costs should be highlighted or even mandated by the registry of charities and monitored when annual accounts are submitted. But even if the charities keep their costs under control, too many are involved in duplicating activities.

Those charities that have a positive net income then face the task of investing their money to the greatest effect, which can be a major challenge with the outcome often being random. The issue is how best to harness the goodwill of donors such that the outcome has a real impact on a disease or other health concern. Here, the large professional charities play a major role in supplementing funds for research and support services that are not met by governments. In most countries, ~12% of funding for research comes from charities. In the UK, that figure is even larger than 20% owing to mega charities such as the Wellcome Trust and Cancer Research UK.

And yet, the strength of small charities close to an individual or family is the emotional power that connects them with their donors. Professional fundraisers motivated only by profit can lose this connection with the cause as they shake a collection box at passers-by. The solution in business would be to merge similar entities and cut costs by sharing the back office. But charities are not businesses and should not be subject to the same hard “bottom line” logic to define their continuation. Partnerships or affiliation with a larger organization would seem like a better option, and those high-profile charities should make it known that they are open to such discussions. In this way, the minnow charity could ask for the funds they raise to be added to other sources to address the problem they are dedicated to. The large organisations have sophisticated processes to identify the best recipient, which would address the question for small operators about how the money should be used most efficiently. In return, the larger charity gains volunteers, funds and enthusiasm from the community. Otherwise, it will take only a few news stories of cases where donations largely go into salaries with little or nothing left for the noble cause to result in spreading cynicism for the essential work of the charity section and less donations being available to address an urgent problem.

Charities are an important manifestation of human generosity, but should not be vanity-driven or self-indulgent projects. Fundraising should do more than support the running of the organization. They need to have a clear plan about what they wish to deliver. Ideally, they should have links in place with a reputable institute, or a larger foundation that will channel their funds to credible projects. And they should be rigorous on maintaining costs to a low level, or else they will, literally, achieve nothing.

## Supplementary information

Peer Review File

