# Supplementary material for: Too much charity?
Source: EMBO Rep. 2025 Jul 29;26(16):3977–8. doi: 10.1038/s44319-025-00542-1 (PMC12373889; doi:10.1038/s44319-025-00542-1)
Supplement: Supplementary file 1 — Peer Review File [file 44319_2025_542_MOESM1_ESM.pdf]

EMBO reports

Too Much Charity?

Frank Gannon

Corresponding author(s): Frank Gannon (FGannon@qimrberghofer.edu.au)

Review Timeline:

Submission Date:	22nd Jul 25
Accepted:	23rd Jul 25

Editor: Holger Breithaupt

Transaction Report: This article type is not peer reviewed.
